# The Cow’s Milk-Related Symptom Score (CoMiSS^™^): A Useful Awareness Tool

**DOI:** 10.3390/nu14102059

**Published:** 2022-05-14

**Authors:** Katerina Bajerova, Silvia Salvatore, Christophe Dupont, Philippe Eigenmann, Mikael Kuitunen, Rosan Meyer, Carmen Ribes-Koninckx, Raanan Shamir, Hania Szajewska, Yvan Vandenplas

**Affiliations:** 1 Department of Pediatrics, University Hospital Brno, Masaryk’s University, 62500 Brno, Czech Republic; bajerova.katerina@fnbrno.cz; 2 Department of Internal Medicine, Geriatrics and Practical Medicine, University Hospital Brno, Masaryk’s University, 62500 Brno, Czech Republic; 3 Department of Paediatrics, University of Insubria, 21100 Varese, Italy; silvia.salvatore@uninsubria.it; 4 Ramsay Group, France et Clinique Marcel Sembat, Paris Descartes University, 92100 Boulogne-Billancourt, France; christophe.dupont@wanadoo.fr; 5 Pediatric Allergy Unit, Department of Pediatrics, University Hospitals of Geneva, 1205 Geneva, Switzerland; philippe.eigenmann@hcuge.ch; 6 Children’s Hospital, Helsinki University Hospital, University of Helsinki, 00029 Helsinki, Finland; mikael.kuitunen@hus.fi; 7 Department Paediatrics, Imperial College London, London SW3 6LY, UK; info@rosan-paediatricdietitian.com; 8 Department Dietetics, Winchester University, Winchester SO23 4NR, UK; 9 Department Medicine, KU Leuven, 3000 Leuven, Belgium; 10 Pediatric Gastroenterology, La Fe University Hospital, 46026 Valencia, Spain; ribes_car@gva.es; 11 Schneider Children’s Medical Center of Israel, Institute of Gastroenterology, Nutrition and Liver Diseases, Petach Tikva 4920235, Israel; raanan@shamirmd.com; 12 Department of Paediatrics, The Medical University of Warsaw, 02-091 Warsaw, Poland; hszajewska@wum.edu.pl; 13 UZ Brussel, KidZ Health Castle, Vrije Universiteit Brussel (VUB), 1090 Brussels, Belgium

**Keywords:** CoMiSS, cow’s milk allergy, food allergy, infant, cow’s milk

## Abstract

The Cow’s Milk-related Symptom Score (CoMiSS™) was developed as a clinical tool aimed at increasing the awareness of health care professionals for the presence and intensity of clinical manifestations possibly related to cow’s milk (CM) intake. This review summarizes current evidence on CoMiSS. We found twenty-five original studies, one pooled analysis of three studies, and two reviews on CoMiSS. Infants exhibiting symptoms possibly related to CM, present with a higher median CoMiSS (6 to 13; 16 studies) than apparently healthy infants (median from 3 to 4; and mean 3.6–4.7; 5 studies). In children with cow’s milk allergy (CMA), 11 studies found that a CoMiSS of ≥12 predicted a favorable response to a CM-free diet; however, sensitivity (20% to 77%) and specificity (54% to 92%) varied. The decrease of CoMiSS during a CM elimination diet was also predictive of a reaction to an oral food challenge to diagnose CMA. A low CoMiSS (<6) was predictive for the absence of CMA. It was shown that no special training is required to use the tool in a reliable way. Intra-rater reliability was high with very low variability (intra-class correlation 0.93; 95% confidence interval 0.90–0.96; *p* < 0.001) in repeated assessments. This review found that CoMiSS cannot be considered as a stand-alone CMA diagnostic tool, but that it is a useful awareness tool for CMA as well as for monitoring symptom improvement.

## 1. Introduction

Health care professionals (HCPs) often see infants presenting manifestations involving the skin (such as eczema, angioedema, urticaria), gastrointestinal (GI) (vomiting, regurgitation, loose and watery stools, constipation, rectal bleeding), respiratory tract (wheezing, chronic cough), and also general symptoms (poor growth, infantile colic and persistent distress) [1]. These symptoms are common and occur in 15–20% of infants [2], but they can also be associated with the diagnosis of cow’s milk allergy (CMA). The reported prevalence of CMA is less than 5.0% [3,4,5]. According to the EuroPrevall data, the prevalence of CMA is even as low as 0.54% (95% confidence interval (CI) 0.41–0.70) [6]. Symptoms occurring up to 2 h after cow’s milk (CM) ingestion are suggestive of an IgE-mediated allergic reaction [7]. In contrast, delayed (2 to 4 weeks after ingestion) and protracted symptoms, mainly affecting the gastrointestinal (GI) tract, may suggest a non-IgE mediated allergy [8]. Whilst most infants outgrow non-IgE mediated CMA by 12 months of age [6], persisting symptoms have also been described after 1 year of age, particularly in a case of food protein-induced enterocolitis syndrome (FPIES) [9].

Differentiating between non-IgE mediated CMA, functional GI disorders (FGIDs), gastro-esophageal reflux disease (GERD), or other (i.e., neurological, metabolic, endocrine, anatomic, and infective) conditions is challenging, as in these conditions many infants present with a combination of symptoms [1,10]. However, for FGIDs, symptoms will per definition be limited to the GI tract and general symptoms, while for CMA also skin and respiratory tract manifestations can be involved. Despite these diagnostic challenges, a timely diagnosis of CMA is crucial to improve faltering growth and quality of life, which may persist even despite effective management [11]. On the other hand, over-diagnosis of CMA and inappropriate association of the abovementioned symptoms with CMA results in unnecessary dietary eliminations, is a burden for caregivers, increases health care costs, and results in possible nutritional deficiencies (i.e., poor growth, micronutrient, and vitamin deficiencies) especially in cases of absent dietetic support [4,12].

A score composed of clinical manifestations reflecting their intensity possibly related to CM-related symptoms was published in 2015 by a group of experts to increase the awareness of HCPs and caregivers for CMA [13]. The authors converted a symptom-based score [14] into the Cow’s Milk-related Symptom Score (CoMiSS^TM^) [13]. CoMiSS is a rapid, short, and easy-to-use tool [15], assessing stool pattern, the presence and intensity of crying and regurgitation, as well as skin and respiratory manifestations (Table 1). The total score ranges from 0 to a maximum of 33. An arbitrary cut-off value ≥12 was selected by consensus to pick up infants at risk of CMA [13]. A CoMiSS above this cut-off is not intended to replace the necessity of the standard diagnostic procedure for CMA, namely a CM elimination diet and oral CM challenge (OFC).

This review aims to summarize available research and evidence for CoMiSS, including its contribution to the diagnosis and management of infants suspected to have CMA.

## 2. Materials and Methods

An electronic search (full-text and abstracts) in NCBI/Pubmed, NCBI/PMC, EBSCO/Academic Search Ultimate, and Ovid/Embase was conducted on 12 December 2021, and updated on 19 February 2022, using the following search terms and keywords: “CoMiSS” and in addition “CoMiSS AND allergy”, with a time period extending back over ten years. Finally, overall, 28 published papers were considered: 25 original studies including more than 3000 infants (22 with data on symptomatic infants, and 3 on presumed healthy infants), 1 pooled analysis (including data of 3 already included studies) and 2 reviews focused on CM symptom-based scores (1 systematic review including 15 original papers on CoMiSS, and the other including 13 original papers included in this review and 10 congress abstracts not considered by this review) (Table 2).

## 3. Results

### 3.1. CoMiSS in Presumed Healthy Infants 

Five studies, including in total 1158 participants [22,23,29,30,32], show that the median (IQR) CoMiSS in healthy infants aged ≤ 6 months varies between 3 (1–5) [22] and 4 (2–7) [29] (Table 3). The percentage of healthy infants with a score ≥12 ranges between 0% (0/94) [23,32] and 4.9% (11/226) [29]. In one study, 13 presumed healthy infants with a score of ≥10 or 8 with a score of ≥12 underwent an open CM challenge, which was positive in 10/13 (76%) cases that scored ≥ 10 and in 7/8 (87.5%) that scored ≥12 [30].

In a study by Vandenplas et al. with participants from four European countries, 563 full-term infants (aged 0–6 months) with no previous drug or dietary treatment were enrolled, showing 3.0 (4) and 3.7 (2.9) as median (IQR) and mean (SD) CoMiSS [22]. The 95th centile in this population scored 9, suggesting that ≥10 would be a logical cut-off to indicate an at-high-risk group for CM associated symptoms [22].

### 3.2. Factors Potentially Affecting CoMiSS in Presumed Healthy Infants

- Country

Overall, CoMiSS in presumed healthy infants was clinically comparable across the four European populations with a 5th centile at 0 to 1, a 50th at 3 to 4, and a 75th at 5 to 6, respectively. Only the 95th centile differed from 8 (Belgium, Italy) to 9 (Spain) and 12 (Poland), respectively [22]. A statistical difference appeared in the median CoMiSS across the participating countries (*p* = 0.002), but with no apparent clinical impact or parental perception of a possible health problem. Post hoc analysis revealed HCPs scoring differently in Poland compared to Belgium (*p* = 0.001), and Poland compared to Italy (*p* = 0.02), possibly in relation to the parental perception of symptoms and the ability to cope. The median score for stools was significantly higher in Poland than in other countries (*p* < 0.001). Median crying and regurgitation scores differed across populations (*p* = 0.001 and *p* = 0.01). Although some statistical differences were observed overall, CoMiSS performed consistently across the four countries.

- Gender

Gender was not associated with statistical differences in the CoMiSS (*p* = 0.76) [22], (*p* = 0.3) [29] or any of the individual symptom scoring components.

- Age (Table 4)

**Figure 1:**
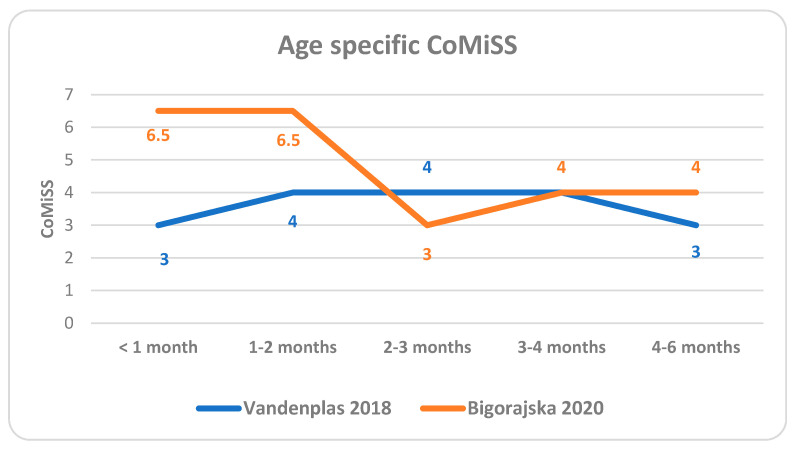
Age specific median CoMiSS.

The influence of age was analyzed in two studies [22,29] (Figure 1). The Vandenplas et al. study [22] (data from four countries) detected a trend towards differences across ages, with higher scores in the 1–2 months and 3–4 months age groups (*p* = 0.09 for overall difference, with *p* values < 0.01 if comparing each two age categories). In particular, a significant decrease with age was seen for crying (*p* < 0.01) and regurgitation (*p* = 0.009). The stool pattern scores did not differ across age categories (*p* = 0.61). In the study carried out in Poland by Bigorajska [29], age had an impact on the total CoMiSS value (*p* < 0.001). However, the number of infants included in some age groups was very small, making the comparison of the outcomes difficult. There is no available data on CoMiSS in healthy infants older than 6 months.

- Breast and formula feeding

Type of feeding did not impact the total CoMiSS in the study by Vandenplas et al. [22], (*p* = 0.43) and in the healthy Spanish cohort assessing inter-rater variability [30]. In contrast, in the Bigorajska study, the type of feeding (exclusive breastfeeding, formula, mixed feeding) significantly influenced CoMiSS (*p* < 0.001) [29]. The median (Q1–Q3) CoMiSS value showed a trend towards being higher, with more prevalent loose stools in breast- than in formula-fed infants (4.0 (2–7) vs. 3.0 (1–4), respectively; *p* = 0.08); however, the difference was not significant.

In the Vandenplas et al. study, type of feeding similarly influenced the stool consistency, with a higher median stool score in exclusively breastfed infants compared to others (2 vs. 0, *p* = 0.02) [22]. Crying, regurgitation, and respiratory symptoms were independent of feeding type.

- Inter-rater and day-to-day variability (Table 5)

Inter-rater variability between HCPs and parents was assessed in Spanish and Belgian infant cohorts [30]. Spanish parents (*n* = 148) of presumed healthy infants, aged 2–6 months, completed the CoMiSS in the waiting room, blinded to the score made by the HCP based on the clinical history provided by the same parents and the physical examination of the infant. The agreement was 75%, 92.6%, and 100% accepting a difference of 0, 1, and 2 points, respectively. In one child, the CoMiSS was 12 according to the parents and 11 according to the HCP.

Among 72 Belgian infants, CoMiSS was recorded by HCPs on day 1 and by parents who were blinded to the HCP score during the 3 following days. The median HCP (IQR) score was 3.0 (5.0) and parental scores were 3.0 (4.0), 3.0 (4.0), and 2.0 (4.0) over the 3 consecutive days. These findings likely reflect the real-life day-to-day variability of clinical symptoms in healthy infants. On day 1, the HCP/parent agreement on the individual subject was 25.1%, which rose to 68.1% with a 2 points tolerance and 77.8% with a 3 points tolerance [30].

### 3.3. CoMiSS in Symptomatic and Allergic Infants

As listed in Table 5, Table 6 and Table 7, twenty-two studies used CoMiSS to assess infants presenting symptoms suspected to be CM related, sixteen studies found in Table 6 and Table 7 (including one pooled analysis [39], including data of 227 subjects from three previously performed studies [14,17,18]) analyzed the evolution of CoMiSS before and during a CM elimination diet in suspected subjects. In seven of these papers (Table 6), authors calculated the sensitivity, specificity, and negative/positive predictive value (NPV/PPV) of CoMiSS in infants with proven CMA. Nine studies (Table 7) used CoMiSS to document the tolerability of a new therapeutic formula by considering the changes or stable values of the score during CM elimination.

Repeated assessment of CoMiSS (Table 5)

Two studies, including overall 142 subjects, were conducted to compare repeated assessments of CoMiSS. The first study (Kozlowska-Jalowska et al.) assessed the CoMiSS in 110 infants (mean age (SD)18.2 (11.7) weeks) with symptoms possibly related to CMA or FGID before the clinical evaluation (retrospective) and 24 h after the medical consultation (prospective) before any intervention [33]. The prospective scores were significantly lower than the retrospective ones (median difference −1.5; 95% CI −2.0 to −1.0; *p* <0.001 (Table 5)). Values exceeded a cut-off ≥12 in 17/110 (15.5%) of the retrospective CoMiSSs versus 11/110 (10.0%) of the prospective data (*p* = 0.1). In the retrospective data, 32/110 (29%) infants exceeded a score > 9, versus 19/110 (17.3%) in the prospective ones (*p* = 0.004). As for the other study by Ursino et al., infants presenting symptoms suggestive of CMA were assessed for inter-rater variability between two HCPs in a South American setting with an interval of 2 to 7 days [36]. The intraclass correlation between the two scores was 0.80 (95% CI 0.63–0.9, *p* < 0.001) [36].

CoMiSS in at-risk infants for cow’s milk associated symptoms (Table 6, Table 7, Table 8 and Table 9)

Comparison between studies is difficult because of differences in inclusion criteria: two studies only considered infants with a score ≥ 12 [16,28], while the other studies considered subjects with any symptom suggestive of being CM associated with no pre-set CoMiSS cut-off [14,15,17,18,19,21,23,25,26,27,32,34,35,37,38].

A cross-sectional study by Selbuz et al. included 168 infants (0–12 months) with suspected CMA (none with rectal bleeding) and a CoMiSS ≥ 12. This study and the resulting cut-off presented a selection bias as infants with CoMiSS < 12 were excluded. Mean ± SD (min-max) CoMiSS at enrolment was 13.6 ± 1.9 (12–22) [28].

In a multicenter, cross-sectional study by Prasad et al., among 83 infants presenting with symptoms possibly related to CMA, 70 (84%) children were diagnosed with CMA (47 with a positive OFC, and the others by Immunocap) [21]. A total of 55 infants out of those 70 (78%) had a score ≥ 12.

In the study by Salvatore et al. enrolling 47 infants aged up to 8 months with persistent unexplained GI symptoms (4 with rectal bleeding), CoMiSS was evaluated before and after 2–4 weeks of a CM elimination diet [23]. Out of the 47 symptomatic infants, 19 (19/47; 40%) were considered as responders to different CM elimination diets. The response to diet was defined by a reduction of the score ≥ 50% and falling under the median of the control population. The median initial CoMiSS value of the responders was 10 (range 8–16), while in the non-responders to diet, the median initial CoMiSS value was 5.5 (range 2–12). The best cut-off score to predict the response to diet was identified as 9, whilst a score <6 was predictive for the absence of CM-related symptoms.

Zeng et al. assessed CoMiSS in 38 Chinese infants suspected of having CMA. The 24 (24/38; 63%) infants with an open OFC-confirmed CMA had a mean (SD) CoMiSS value of 7.4 (±2.3); 10/24 (42%) had positive IgE tests, 15/24 (62.5%) were presenting bloody stools. In the other 14 (14/38; 37%) infants, the mean (SD) CoMiSS was significantly lower (4.1 (±1.6) (*p* < 0.05) [25].

CoMiSS was determined in 112 Turkish infants in a study by Sirin Kose et al.: 49/112 (44%) infants with CMA confirmed by OFC or positive IgE (>5 kU/L) and positive skin prick test; 39/112 (35%) with hen’s egg allergy; and 24/112 (21%) with combined CMA/hen’s egg allergy [26]. Median (Q1–Q3) initial CoMiSS value was 13 (10.5–16) in CMA, 12 (7–14) in hen’s egg allergy, and 13.4 (12–17.8) in CMA–hen’s egg allergy. After a CM and/or hen’s egg elimination diet, the mean (±SD) CoMiSS decreased significantly in all groups (*p* = 0.009). Infants diagnosed with isolated CMA presented with a pre-elimination diet score > 9 in 88% (43 patients) and ≥ 12 in 69% (34 patients). The initial score decreased on the elimination diet by at least 50% in 84% of subjects.

In a retrospective study in 40 children with CMA by Balasa et al., 24/40 subjects (60%) with IgE-mediated CMA had a CoMiSS score ≥ 12, and 16/40 subjects (40%) with non-IgE-mediated CMA scored between 8 and 11 [27]. The authors did not specify the methodology of how CoMiSS was assessed and also did not report the median of CoMiSS at enrolment or the age of enrolled subjects.

El Desouky et al., reported a CoMiSS value > 12 in 35.8% (43/120) of Egyptian infants aged up to 18 months with gastrointestinal, cutaneous, and respiratory symptoms suggesting CMA [34]. All of these 120 infants underwent OFC and CMA was diagnosed in 44 cases (36%). However, the authors did not determine clearly which CoMiSS cut-off they used, and the complete data set to determine the sensitivity and specificity of the CoMiSS cut-off were missing.

In a 2021 multicenter real-life study by Vandenplas et al. including 268 infants referred for presumed CMA, the mean CoMiSS value was 11.1 (range 6.5 to 16) and the median was 11.0; 72% of subjects had a CoMiSS value of >9 and 49% a CoMiSS ≥ 12 [15]. The overall mean and median of CoMiSS values decreased to 4.2 and 4.0, respectively, on dietary intervention. Interpretation of data from this study should be cautious as CMA was not confirmed with an OFC.

A large trial in Chinese infants, the MOSAIC study, aimed to determine the optimal cut-off of CoMiSS to test if CoMiSS could subsequently be considered as a stand-alone diagnostic tool for CMA [37]. A total of 299 enrolled infants (<6 months old) had symptoms possibly related to CMA; CoMiSS at baseline and after two weeks of CM elimination with an amino-acid formula (AAF) were assessed in 254 infants. A total of 250 infants underwent an open OFC, which was positive in 217 (88%). The CoMiSS median (IQR) at inclusion was higher in infants with positive challenge (8 (6–11)) than in the negative ones (5 (4–10)). Median (IQR) CoMiSS decrease was more pronounced in the positive group (−3 (−6 to −1)) than in the negative group (−2 (−5 to −1)).

In the studies by Dupont et al., Fierro et al., and Rossetti et al., the authors used CoMiSS as a tracking tool to demonstrate the stable low CoMiSS after introducing the new test formula in infants already diagnosed with CMA responding to an elimination diet [20,24,31].

- Response to cow’s milk elimination

The effect of an elimination diet lasting 1 to 4 weeks on CoMiSS values was assessed in 12 studies [14,15,16,17,18,19,23,26,28,37,38,39]. In most of the studies, the decrease of CoMiSS in infants with a positive challenge test was larger than in the group with a negative OFC. In infants with suspected but not proven CMA, the mean (SD) CoMiSS decrease following elimination varied from −5.9 (3.2) [18] to −6.5 (4.5) [19]. In the study by Selbuz et al. [28], CoMiSS values after the elimination diet decreased by 7 points (95% CI −6.6 to −7.5 *p* = 0.001); 59% of infants with a score decrease ≥3 following elimination diet had a positive OFC. The study by Sirin Kose et al. [26] on infants with CMA, hen’s egg allergy, or combined allergy, classified 112 infants according to IgE status (IgE-positive, IgE-negative, and mixed). Scored after the elimination diet, CoMiSS decreased to 4.0 (1.8–6.0) in IgE-positive, 3.5 (1.0–6.0) in IgE negative, and 3.5 (2.0–6.0) in the mixed group. This study suggests that CoMiSS can reflect the evolution of symptoms even in different types of food allergies.

In the study by Vandenplas in formula-fed infants, the decrease of CoMiSS was larger in infants receiving an AAF showing a median (min; max) −10 (−27; −1) and a mean (SD) −9.5 (4.5) compared to those on a eHF reporting a median (min; max) −6 (−19; 5) and a mean (SD) −6.4 (5.1) [15]. In Salvatore’s study, the median decrease on AAF compared with eHF was −8.5 vs. −4.5, respectively [23].

In nine studies, CoMiSS symptoms were assessed separately at inclusion after a period of a CM elimination diet or repeated according to the study protocol (Table 8) [14,17,18,19,20,23,26,35,37].

- Sensitivity and specificity

An awareness tool should, as a priority, have high sensitivity and be accompanied by high specificity. The sensitivity, specificity, and positive and negative predictive values (PPV, NPV) were calculated in seven studies [21,23,25,26,28,34,37] (Table 6). As inclusion criteria and design differed among the trials, drawing a general conclusion is speculative. The discrepancy in cut-off values, ranging from ≥5.5 to ≥12, can be explained by the differences in study design: while some studies used a CoMiSS above a specific cut-off as an inclusion criterion [16,28], other studies used symptoms as an inclusion criterion and determined CoMiSS as additional information [21,23,25,34,37]. The sensitivity and specificity of CoMiSS reached 70% or more, and up to 90% in some studies. The range of values suggests that CoMiSS may operate differently according to study design and type of symptoms presented. The reduction of mean and median CoMiSS to <6 points was associated with clinical response to the diagnostic elimination diet [14,15,16,17,18,19,23,26,28,37,39].

The review by Calvani et al. included 23 papers and congress abstracts that have evaluated CoMiSS and found 12 reporting a significant reduction in CoMiSS after an elimination diet [41]. Moreover, a reduction of >50% was predictive of a subsequent positive OFC. The authors concluded that CoMiSS is a valuable tool to aid the diagnosis of CMA, especially in non-IgE mediated allergy, but further validation is still needed before it can be used routinely in clinical practice. Five studies [21,23,25,28,37] (four of those included in the review by Calvani [41]) assessed the receiver operating characteristic curve (ROC-a tool for evaluating and optimizing a binary classification system/test, which shows the relationship between the specificity and sensitivity of a given test) and calculated an area under the curve (AUC) to determine the best diagnostic cut-off (Table 6).

One study [37], not included in the other review [41], calculated sensitivity and specificity (CMA proven by OFC) of the ≥50% reduction of baseline CoMiSS. Such an approach improved the specificity but at the expense of sensitivity (Table 9).

A systematic review by Thompson et al. [40], including 15 diagnostic-accuracy studies [14,16,17,18,20,21,22,23,24,25,26,28,29,30,42], considered that there is no well-defined diagnostic role for symptom-based scores, including CoMiSS, and that current estimates of their accuracy should be interpreted with caution.

### 3.4. CoMiSS in Conditions Other Than CM Allergy

Vandenplas published in 2021 a prospective real-life study evaluating the evolution of CoMiSS in 196 infants (aged 0–4 months) who were presenting functional gastrointestinal symptoms (crying, regurgitation, and stools evaluated by CoMiSS; the sum of two symptoms out of three were >4) treated with a partial whey hydrolysate and containing other functional components (e.g., pre- and probiotics) [35]. The inclusion criterion was CoMiSS > 4 since 4 was reported to be the median value in presumed healthy infants. The efficacy of the formula was documented by a decrease of CoMiSS values from baseline mean (SD) 6.5 (3.1) to 4.9 (3.1) on day 14. Since a partial hydrolysate was effective in these infants with a mean (SD) CoMiSS of 6.5 (3.1) at inclusion, these data suggest that infants with this CoMiSS value are unlikely to have CMA.

Petriashvili et al. assessed 68 Georgian children aged up to 2 years old with CoMiSS. A total of 11 out of 68 (16.2%) children presented with mild course of AD (SCORAD < 20) and mean (SD) CoMiSS 7.7 (3.0), 63.2% (43/68) had moderate course AD (SCORAD 20–40) and mean (SD) CoMiSS 7.3 (3.9), and 20.6% (14/68) had a severe course of AD (SCORAD > 40) with mean (SD) CoMiSS 11.3 (5) [32]. These findings suggest that infants with a severe course of AD should be considered at risk of CMA.

## 4. Discussion

This review brings an updated summary of current evidence for CoMiSS, including its contribution to the diagnosis and management of infants with CMA and its performance in healthy and symptomatic populations. It was shown that no special training is required to use the tool in a reliable way. Intra-rater reliability is high with very low variability (intra-class correlation 0.93; 95% confidence interval 0.90–0.96; *p* < 0.001) in repeated assessments.

An accurate diagnosis of CMA and early detection of CM-related symptoms is pivotal to avoid persisting symptoms, nutritional deficiencies, and impaired quality of life [4,12]. However, identifying these infants is often challenging for HCPs, particularly when facing infants with a spectrum of different clinical manifestations, frequently reported in this age group, and negative IgE testing. In subjects with a score ≥ 12, a significant reduction of CoMiSS is observed on a CM elimination diet, regardless of the clinical presentation and IgE level [14,16,17,18,19,23,26,28,35,37,38,39]. HCPs who used CoMiSS in their daily routine found it a helpful and fast-to-use tool [15].

The decrease of CoMiSS in symptomatic infants on an elimination diet raised the need to define the value of CoMiSS in apparently healthy infants. In an assessment of 563 presumed healthy infants 0 to 6 months old in Spain, Italy, Poland, and Belgium, CoMiSS was defined by the 95th centile to be 9 [22]. Interestingly, in presumed healthy infants included in a Spanish subgroup [30], CMA was diagnosed in 7/8 (87.5%) with a CoMiSS ≥ 12 and in 10/13 (76%) with a CoMiSS ≥ 10. These findings suggest that some infants are not considered symptomatic by caregivers, but a high CoMiSS may indicate the presence of CMA. The low median CoMiSS in presumed healthy infants and the decrease of CoMiSS in CM allergic infants, seen after dietary intervention, indicate that CoMiSS < 6 is predictive for the absence of CMA.

A cut-off of ≥9 according to the obtained ROC has recently been proposed, showing 84% sensitivity, 85% specificity, 80% PPV, and 88% NPV for a clinical response to a CM elimination diet [23].

Two studies in formula-fed infants documented larger decreases of CoMiSS in infants receiving an AAF compared to those with eHF [15,23]. The smaller reduction of CoMiSS with eHF than with AAF may be related to the different residual peptide size of eHFs, and presumed remaining potential to cause symptoms of allergic reactions in sensitized individuals [43]. For some eHFs, clinical effect failure rates above 20% have been reported [44]. However, one study did not provide information on the eHFs used [15]. The larger reduction of CoMiSS values on AAF than on eHF could also reflect the higher initial score in the most severe cases selected to start an elimination diet, but this approach was not statistically analyzed and documented.

The strength of this review is that it includes recently published and to date unreviewed studies [15,32,33,34,35,36,37,38]. It summarizes data on symptomatic and presumed healthy infants and assesses studies with the intent of showing the modality of CoMiSS use in different clinical settings, and also its usefulness as an effective symptom-tracking tool. The awareness role of CoMiSS in recognizing infants at possible risk of CMA is well documented. We have reviewed and systematically tabled CoMiSS according to types of included populations (healthy, symptomatic, CM allergic), as well as the changes of CoMiSS during CM free diet and how each of the CoMiSS items reflect the trigger elimination (Table 8). Finally, we found that the reviewed data does not allow CoMiSS to be used as a standalone diagnostic tool even when considered in comparison to previous reviews [40,41].

This review points out some current limitations with existing data. Data in presumably healthy infants from outside Europe or in age groups beyond 6 months of age are still missing. Since CoMiSS does not include all symptoms associated with CMA, some infants with CMA will have a low CoMiSS before or during an elimination diet. In particular, angio-edema, anaphylaxis, failure to thrive, and rectal bleeding are not part of the symptoms listed in CoMiSS. Two studies evaluating CoMiSS had many infants with rectal bleeding as a major symptom, showing recovery during an elimination diet [25,37]. However, hematochezia is commonly seen in infants, and CMA is not the only cause of this symptom. A specific diagnostic work up according to presence of other clinical conditions (i.e., infection, anal fissure or a rectal prolapse) is recommended [45,46].

Blinding when assessing CoMiSS was only mentioned in four papers [23,30,36,37]. A selection bias was introduced in the studies enrolling only infants with scores ≥ 12 (not including infants with CMA with a lower score) and in others not performing OFC in all responsive subjects [28]. Moreover, a control group was seldom included [23,32].

Fourteen studies used CoMiSS to assess infants suspected of suffering from CMA [14,15,16,18,19,21,23,25,26,28,33,34,36,37]. However, these studies exhibited an important heterogeneity of enrolled subjects, inclusion criteria, study design, and outcome measures so that a meta-analysis is not feasible [40].

A very low variability was observed when CoMiSS was scored by HCPs, parents prospectively over three days by the same rater, or by two different HCPs [30,33,36]. Hence, CoMiSS has the potential to become an effective tool in monitoring infants on nutritional intervention in the growing era of virtual medicine and mobile applications. However, it warrants caution during the diagnostic process because parents may misinterpret symptoms of other, potentially serious, conditions (i.e., infections, neurological or surgical disorders). Therefore, although parents can fill in the CoMiSS in a reliable way, supervision by HCPs remains mandatory.

In pediatric clinical practice, the important contribution of a validated and standardized score, as for instance, the SCORAD [47] and the Pediatric Crohn’s Disease Activity Index (PCDAI) [48] is well recognized. Both reflect the evolution of symptoms and help clinicians analyze the effectiveness of treatment in atopic dermatitis and Crohn’s disease. CoMiSS could be considered as a tool for evaluating the response to an elimination diet and the potential reaction during an OFC.

It may be of interest to consider the future development of tools for more specific diagnoses such as FPIES, allergic proctocolitis, eosinophilic esophagitis, lactose intolerance, or eczema aggravated by non-IgE mediated dairy allergy. Furthermore, soy co-reactivity in combination with CM is common in proctocolitis and FPIES and should be considered in upcoming versions of an awareness tool.

At present, two other tools with the intention of helping to diagnose CMA have been published, each with one study [42,49]. Gibbons et al. retrospectively tested a questionnaire consisting of 25 yes/no questions in 43 children aged up to 2 years old [42]. The authors declared a sensitivity of 88% and specificity 71% for a cut-off of 6, improving to 79% and 93% if only those statistically important items were considered. Muñoz-Urribarri et al. proposed 16 yes/no questions in infants and children up to 5 years of age and showed that a cut-off of 7 reached a sensitivity of 94.4% and a specificity of 96.9% [47].

## 5. Conclusions

As originally intended, CoMiSS is an easy-to-use practical awareness tool for evaluating cow’s milk-related symptoms. Benefits of CoMiSS include symptom tracking before and during an elimination diet used for the management of CMA. Particularly, a high baseline CoMiSS associated with a significant reduction during a CM elimination diet is specific and supports the diagnosis of CMA. However, at present, CoMiSS cannot be considered as a stand-alone diagnostic tool for CMA. The pros and cons of updating CoMiSS, reducing the cut-off or including new items, are a matter of current debate.

## Figures and Tables

**Table 1:** CoMiSS^TM^.

Crying				
≤1 h/day	0			
1 to 1.5 h/day	1			
1.5 to 2 h/day	2			
2 to 3 h/day	3			
3 to 4 h/day	4			
4 to 5 h/day	5			
≥5 h/day	6			
Regurgitation				
0 to 2 episodes/day	0			
≥3 to ≤5 of small volume	1			
>5 episodes of >1 coffee spoon	2			
>5 episodes of ± half of the feed in <half of the feedings	3			
Continuous regurgitations of small volume >30 min after each feeding	4			
Regurgitation of half to complete volume of a feeding in at least half of the feedings	5			
Regurgitation of the “complete feeding” after each feeding	6			
Stools (Bristol scale)				
Type 1 and 2 (hard stools)	4			
Type 3 and 4 (normal stools)	0			
Type 5 (soft stools)	2			
Type 6 (liquid stools, if unrelated to infection)	4			
Type 7 (watery stools)	6			
Skin Symptoms	absent	mild	moderate	severe
Atopic eczema				
Head, neck, and trunk	0	1	2	3
Arms, hands, legs, and feet	0	1	2	3
Urticaria	no	yes		
	0	6		
Respiratory symptoms				
No respiratory symptoms	0			
Slight symptoms	1			
Mild symptoms	2			
Severe symptoms	3			

**Table 2:** Included studies.

1st Author [Ref]	Year	Type of Study *	Sample Size Included (PP)	Title	Conflict of Interest
Vandenplas [14]	2013	observation prospective	116 (85)	Treating cow’s milk protein allergy: a double-blind randomized trial comparing two extensively hydrolyzed formulas with probiotics.	yes
Vandenplas [16]	2014	validation prospective	116 (84)	A pilot study on the application of a symptom-based score for the diagnosis of cow’s milk protein allergy.	yes
Vandenplas [17]	2014	observation prospective	40 (36)	Safety and tolerance of a new extensively hydrolyzed rice protein-based formula in the management of infants with cow’s milk protein allergy.	yes
Vandenplas [18]	2014	observation prospective	72 (52)	Extensive protein hydrolysate formula effectively reduces regurgitation in infants with positive and negative challenge tests for cow’s milk allergy.	yes
Vandenplas [19]	2016	observation prospective	71 (50)	Safety of a thickened extensive casein hydrolysate formula.	yes
Dupont [20]	2016	observation prospective	30	Tolerance and growth in children with cow’s milk allergy fed a thickened extensively hydrolyzed casein-based formula.	yes
Prasad [21]	2018	validation prospective	83	Cow’s milk-related Symptom Score as a predictive tool for cow’s milk allergy in Indian children aged 0–24 months.	no
Vandenplas [22]	2018	observation prospective	891 (563)	The Cow Milk Symptom Score (CoMiSS^TM^) in presumed healthy infants.	yes
Salvatore [23]	2019	validation prospective	47	Testing the cow’s milk-related symptom score (CoMiSS) for the response to a cow’s milk-free diet in infants: a prospective study.	no
Rossetti [24]	2019	observation prospective	30 (29)	Hypoallergenicity of a thickened hydrolyzed formula in children with cow’s milk allergy.	no
Zeng [25]	2019	validation prospective	38	Assessment of Cow’s milk-related symptom scores in early identification of cow’s milk protein allergy in Chinese infants.	no
Sirin Kose [26]	2019	validation prospective	49 CMA 39 HEA 24 CMA + HEA	The efficiency of the symptom-based score in infants diagnosed with cow’s milk protein and hen’s egg allergy.	no
Balasa [27]	2019	observation retrospective	40	Assessment of IgE-Mediated and Non-IgE-Mediated Cow’s Milk Protein Allergy in Children.	no
Selbuz [28]	2020	validation prospective	168	Assessment of cow’s milk-related symptom scoring awareness tool in young Turkish children.	no
Bigorajska [29]	2020	observation prospective	226	Cow’s Milk-Related Symptom Score in Presumed Healthy Polish Infants Aged 0–6 Months.	no
Vandenplas [30]	2020	observation prospective	220 (Spain 148 Belgium 72)	The Cow’s Milk-Related Symptom Score (CoMiSS^TM^): Health Care Professional and Parent and Day-to-Day Variability.	yes
Fierro [31]	2020	observation prospective	41 (30)	A well-tolerated new amino acid–based formula for cow’s milk allergy.	yes
Petrashvili [32]	2020	validation prospective	68	The peculiarities of clinical course of atopic dermatitis and the comorbid conditions in early infancy.	no
Kozlowska–Jalowska [33]	2021	observation prospective	110	Retrospective and prospective determination of the Cow’s Milk-related Symptom Score (CoMiSS™) values in symptomatic infants.	no
El Desouky [34]	2021	observation prospective	120	Assessment of CoMiSS among children with cow’s milk allergy at Zagazig University Hospital.	no
Vandenplas [35]	2021	observation prospective	196 (171)	An observational real-life study with a new infant formula in infants with functional gastro-intestinal disorders.	yes
Vandenplas [15]	2021	observation prospective	268 (208)	How are infants suspected to have cow’s milk allergy managed? A real world study report.	yes
Ursino [36]	2021	observation prospective	32	Cultural adaptation and validation of the Spanish version of the Cow’s Milk-related Symptom Score (CoMiSS) for cow’s milk protein allergy.	no
Vandenplas [37]	2022	validation prospective	299 (250)	Assessment of the Cow’s Milk-related Symptom Score (CoMiSS) as a diagnostic tool for cow’s milk protein allergy- A prospective, multicenter study in China (MOSAIC study).	yes
Vandenplas [38]	2022	validation prospective	194 (137)	Effects of an extensively hydrolyzed formula supplemented with two human milk oligosaccharides on growth, tolerability, safety and infection risk in infants with cow’s milk protein allergy: a randomized, multicenter trial.	yes
Vandenplas [39]	2017	validation	170	Pooled analysis of the Cow’s Milk-related-Symptom-Score (CoMiSS™) as a predictor for cow’s milk related symptoms.	yes
Thompson [40]	2021	systematic review	15 studies	Symptom scores in the diagnosis of pediatric cow’s milk protein allergy: A systematic review.	no
Calvani [41]	2020	review	13 studies and 10 congress abstracts	Non-IgE- or mixed IgE/non-IgE-mediated gastrointestinal food allergies in the first years of life: old and new tools for diagnosis.	no

Legend: CMA—cow’s milk allergy; HEA—hen’s egg allergy; * validation-testing CoMiSS as an outcome, observation using CoMiSS as a symptom validation.

**Table 3:** CoMiSS in presumed healthy infants.

Publication [Ref]	Country F/M	Type of Milk FF/BF/FF + Bf at Enrollment	Gestational Age	Age at Enrolment Median (IQR)	CoMiSS Median (IQR)	CoMiSS Mean ± SD	P 95	CoMiSS ≥ 12 N (%)	CoMiSS >9 N (%)
Vandenplas [22]	Belgium Italy Spain Poland	204/283/76	on term	8.7 (1.9) weeks	3 (1–5)	3.7 ± 2.9	9	9 (1.5%)	28 (5) %
Bigorajska [29]	Poland	34/176/16	median (IQR) 39 (39–40) weeks	4 (3–4) months	4 (2–7)	4.7 ± 2.9	11	11 (4.9) %	n.r.
Salvatore [23]	Italy	n.r.	n.r.	3 (n.r) months	3 (0–11) (min-max) IQR n.r.	n.r.	n.r.	0	1(1.1%)
Vandenplas Spanish cohort [30]	Spain	n.r.	n.r.	2.3 (2.9) months	n.r.	n.r.	n.r.	7 (4.7%)	11(7.4%)
Vandenplas Belgian cohort [30]	Belgium	n.r.	n.r.	3 (0.5) months	3.7 (5.0)	n.r.	n.r.	1 (1.4%)	1(1.4%)
Petriashvili [32]	Georgia	n.r.	n.r.	up to 2 years	n.r.	3.6± 1.8	n.r.	0%	n.r.

Legend: n.r.—not reported; P: percentile.

**Table 4:** Age specific CoMiSS in presumed healthy infants.

	Age	No	Min	P05	P25	Median	P75	P95	Max
V	<1 mo	139	0	0	1	3	5	8	10
B	1 mo	28	3	3	5	6.5	9	13.3	15
V	1–2 mo	129	0	0	2	4	6	10	14
B	2 mo	22	0	1	4	6.5	8.8	11	12
V	2–3 mo	94	0	0	2	4	6	9	10
B	3 mo	55	0	0	1	3	5	9.3	14
V	3–4 mo	88	0	0	1	4	6	11	15
B	4 mo	72	0	0	2	4	7	11.5	15
V	4–6 mo	113	0	0	1	3	5	8	12
B	5 mo	34	0	1.3	3	4	6	9	10
B	6 mo	15	0	0	0.5	4	4.5	9	9

Legend: B—Bigorajska [29]; V—Vandenplas [22].

**Table 5:** Variability of CoMiSS in repeated assessment.

Publication [Ref] Clinical Presentation	Age	Inter-rater Variability (IV)	No. of Subjects	Repetition Variability (RV)	Total Scores Compared (IV)	Total Scores Compared (RV)	CoMiSS ≥ 12 N	CoMiSS ≥ 10	Conclusion
Vandenplas Spanish cohort [30] PH	median (IQR) 2.3 (2.9) mo	HCP vs. parent	148 vs. 148	no	ICC 0.981 95% CI 0.974–0.986 *p* < 0.001	n.r.	7/8 HCP/parent	11/12 HCP/parent	excellent agreement
Vandenplas Belgian cohort [30] PH	median (IQR) 3 (0.5) mo	HCP vs. parent *°	72 vs. 72 ° 72 vs. 72 vs. 72 °°	°° parent 3 times on 3 consecutive days **	ICC 0.53 95% CI 0.34–0.68 *p* < 0.001	ICC 0.93 95% CI 0.90–0.96 *p* < 0.001	n.r.	n.r.	* moderate ICC estimate ** excellent ICC estimate
Kozlowska-Jalowska [33] Symptomatic	mean (±SD) 18.2 (±11.7) weeks	no	110 vs. 110	1 HCP retrospective vs. prospective	n.r.	MD−1.5 95% CI −2.0 to −1.0 *p* < 0.001	17/11 (*p* = 0.109) retrospective vs. prospective	32/19 (*p* = 0.004) retrospective vs. prospective	scores determined retrospectively and prospectively differed
Ursino [36] Symptomatic	median (IQR) 3 (2) mo	HCP 1 vs. HCP 2	32 vs. 32	no	ICC 0.80 95% CI 0.63–0.9 *p* < 0.001.	n.r.	n.r.	n.r.	substantial ICC estimate

Legend: ICC—intra-class correlation coefficient; MD—median difference; n.r.—not reported; IV—inter-rater variability (IV); RV—repetition variability; PH—presumed healthy; ° inter-rater variability HCP vs. parent; °° repetition variability parent 3 consecutive days; * HCP vs.parent variability ICC estimate; ** repetition variability parent 3 consecutive days ICC estimate.

**Table 6:** Validation studies on CoMiSS in CMA suspected and CM allergic infants.

Publication [Ref]	No. of Subjects (PP)	Age at Enrolment	Baseline CoMiSS Mean ± SD (min-max)	Elimination Period	Mean ± SD (min-max) After Elimination CMA+	Mean ± SD (min-max) After Elimination CMA−	Cut-Off	AUC	Sensitivity	Specificity	PPV	NPV
Prasad [21] °	83	12.5 ± 6.4 w mean ±SD	16.2 ± 6.8 (2–32)	15 days	n.r.	n.r.	≥12	0.68	77%	66%	93%	33%
Zeng [25] °	38	1–6 mo: 33 7–12 mo: 5 (2–12 mo) (min-max)	7.4 ± 2.3 (CMPA +)	4 weeks	n.r.	n.r.	5.5	0.89	87.5%	78.6% **	n.r.	n.r.
Salvatore [23] °	47	median 3 (10 d–8 mo) (min-max)	8 (2–16) median (min-max)	2–4 weeks	2 (n.r.) median (IQR)	n.r.	≥9 {≥12}	0.91 {n.r.}	84% {37%}	85% {92%}	80% {77%}	88% {68%}
Selbuz [28] ° CoMiSS ≥12 and decrease after elimination ≥3	168	87 (16–330) days (limit n.r.)	13.6 ± 1.9 (12–22)	4 weeks	5.8	5.9	12.5	0.57	64.8%	54.4%	n.r.	n.r.
Vandenplas [37] °	299 (250)	16.1 (9.9–20.8) median (IQR)	8 (5–10) median (IQR); 0–24 (min-max)	2 weeks	5 (3–7) median (IQR)	3.5 (2–7) median (IQR)	≥6 {≥12}	0.67 {n.r.}	78.8% {20.3%}	51.5% {87.9%}	91.4% {91.7%}	27.0% {14.4%}
El Desouky [34] °	120	6.60 ± 4.82 mo mean ± SD	11.2 ± 2.8 (n.r.-n.r.)	n.r.	n.r.	n.r.	>12	n.r.	86.4%	93.4%	88.3%	92.2%
Sirin Kose [26] °	49	4.7 ± 1.9 mo mean ± SD ***	13 (5) median (IQR)	4 weeks	4 (4) median (IQR)	n.r.	≥10 {≥12}	n.r. n.r.	87,8% {69,4%}	n.r.	n.r.	n.r.
Vandenplas [16] ° CoMiSS ≥12	116 (84)	72 (53–122) days median (IQR) ***	13.65 ± 1.75 (12–21)	4 weeks	5.12 ± 3.39 (0–18)	6.81 ± 3.01 (1–13)	≥12	n.r.	n.r.	n.r.	n.r.	n.r.
Balasa [27] *	40	n.r.	n.r.	n.r.	n.r.	n.r.	≥12	n.r.	n.r.	n.r.	n.r.	n.r.
Vandenplas [15] °	268 (208)	18,4 (1.4–80.6) w median (min-max)	11.1± n.r. median 11.0	3 weeks	4.2 after elimination		n.r.	n.r.	n.r.	n.r.	n.r.	n.r.
Vandenplas [39] °	170	86 (60–122) median (Q1–Q3)	13 (12–15) median (Q1–Q3)	1 month	5(3–7) median (Q1–Q3)		n.r.	n.r.	n.r.	n.r.	n.r.	n.r.

Legend: PP—per protocol; ° including infants with clinical suspicion of CMA; * including infants with proven CMA; ** 95%CI: 0,722, 0,978; *** at diagnosis; n.r.—not reported; PPV—positive predictive value; NPV—negative predictive value; AUC—area under the curve.

**Table 7:** CoMiSS as a validation tool of efficacy and safety in studies on new treatment formulas.

Publication [Ref]	Inclusion Criteria	Age at Inclusion	CoMiSS Before Treatment Initiation	Elimination Period	Follow Up Period with Treatment Formula	CoMiSS During Treatment
Vandenplas [14]	Suspicion of CMA	median (IQR) days eWHF 80 (57–136) eCHF 64 (48–114)	mean ± SD (range) eWHF 13.58 ± 2.20 (5–21) eCHF 13.79 ± 1.47 (12–17)	1 mo	10 months	mean ± SD 1 mo: 5.16 ± 3.16 2 mo: 3.98 ± 2.92 4 mo: 2.79 ± 2.63 6 mo: 2.11 ± 2.17 8 mo: 1.33 ± 1.79 10 mo: 1.04 ± 1.02
Vandenplas [17]	CMA proven before inclusion- positive challenge with CMP	mean ± SD months 3.4 ± 1.5 median (range) 3 (0–6) months	mean ± SD 13.50 ± 5.2	1 mo	6 months	mean ± SD 1 mo: 3.5 ± 2.3 3 mo: 2.4 ± 1.9 6 mo: 1.5 ± 2.0
Vandenplas [18]	Suspicion of CMA	mean ± SD days total 87.5 ± 46.20 TeCHF 80.00 ± 44.00 NTeCHF 94.7 ± 47.7	mean ± SD total 14.1 ± 3.5 TeCHF 14 ± 3.6 NTeCHF 14.1 ± 3.4 CMA+ 14.3 ± 3.4 CMA- 13.9 ± 3.8	1 mo	6 months	decrease after 1 mo mean ± SD total: −7.4 ± 5.5 TeCHF: −7.7 ± 5.2 NTeCHF: −7.2 ± 5.7 CMA+: −8.6 ± 5.3 CMA-: −5.9 ± 3.2
Vandenplas [19]	Suspicion of CMA	mean ± SD days total 90.51 ± 49.02 TeCHF 80.77 ± 43.17 NTeCHF 99.97 ± 43.17	mean ± SD total 14.1 ± 3.5 TeCHF 14 ± 3.6 NTeCHF 14.1 ± 3.5 CMA+ 14.3 ± 3.4 CMA? 13.9 ± 3.8	1 mo	6 months	decrease after 1 mo mean ± SD Total: −7.5 ± 5.2 TeCHF: −7.6 ± 5.2 NTeCHF: −7.4 ± 5.3 CMA+: −8.4 ± 5.2 CMA?: −6.5 ± 4.5
Dupont [20]	CMA proven before inclusion	mean ± SD months 4.8 ± 3.0	n.r.	n.r.	120 days	mean ± SD Day 0: 7.4 ± 4.4 Day 14: 3.2 ± 2.3
Rossetti [24]	CMA proven before inclusion	mean ± SD months 8.03 ± 7.43 median (range) 6 (1–31) months	n.r.	n.r.	3 months	mean ± SD Day 0: 1.4 ± 2.0 Day 7: 0.7 ± 1.2
Fierro [31]	CMA proven before inclusion	mean ± SD months 2.1 ± 2.52	n.r.	n.r.	1 week	mean ± SD Visit 1: 1.37 ± 1.59 Visit 4:0.75 ± 0.55
Vandenplas [35]	FGID	mean ± SD months 1.5 ± 1.0 median (Q1–Q3) 1.1 (07–2.1) months	mean ± SD (range) 6.46 ± 3.09 (0–15) median (Q1–Q3) 6 (4–8)	7 d	2 weeks	mean ± SD day 3: 5.21 ± 2.90 day 7: 4,98 ± 2.93 day 14: 4.92 ± 3.06
Petriashvili [32]	AD	up to 2 years	SCORAD mean (SD) <20 7.7 (3.0) 20–40 7.3 (3.9) >40 11.3 (5.0)	n.r.	n.r.	n.r.
Vandenplas [38]	CMA	mean ± SD months test formula 3.2 ±1.7 control formula 3.2 ± 1.7	test formula 12.08 (95%CI, 10.75–12.63) control formula 11.65 (95% CI, 10.75–12.63)	1 mo	6 months (follow up n.r.)	1 mo mean test formula 3.38 (95% CI, 1.91–2.69) control formula 2.73 (95%CI, 1.42–291)

Legend: n.r.—not reported.

**Table 8:** Evolution of CoMiSS items before and after elimination period.

**Publication [Ref]**	**Group**	**Crying** **Score**	**Crying Score**	***p***	**Regurgitation Score**	**Regurgitation Score**	***p***	**Stool** **Score**	**Stool** **Score**	***p***
		**Mean (SD)**	**Mean (SD)**		**Mean (SD)**	**Mean (SD)**		**Mean (SD)**	**Mean (SD)**	
		**Baseline**	**After CM** **Elimination**		**Baseline**	**After CM Elimination**		**Baseline**	**After CM** **Elimination**	
Sirin Kose [26]	CMA+	median (Q1–Q3) 1 (0–4.5)	n.r.	n.r.	median (Q1–Q3) 1 (0–4)	n.r.	n.r.	median (Q1–Q3) 4 (4–6)	n.r.	n.r.
Salvatore [23]	CM-free diet responders	2.7 (1.9) *	n.r.	n.r.	2.3 (1.9) **	n.r.	n.r.	2.6 (1.9) ***	n.r.	n.r.
Salvatore [23]	CM-free diet non -responders	2.1 (1.9) *	n.r.	* NS	1.8 (1.9) **	n.r.	** NS	0.78 (1.8) ***	n.r.	*** NS
Vandenplas [14]	eWHF	4.2 (2.0)	1.1 (1.4)	n.r.	2.6 (1.7)	1.1 (1.2)	n.r.	3.5 (1.5)	1.8 (1.8)	n.r.
Vandenplas [14]	eCHF	4.6 (1.8)	1.5 (1.7)	n.r.	3.0 (1.6)	1.8 (1.2)	n.r.	3.5 (1.3)	1.4 (1.6)	n.r.
Vandenplas [17]	CMA+	3.8 (2.0)	0.5 (0.8)	<0.001	2.4 (2.2)	0.6 (0.9)	< 0.001	normal/ abnormal 5.3%/94.7%	normal/ abnormal 52.6%/47.4%	<0.0001
Vandenplas [18]	CMA+	3.7 (2.3)	1.1 (1.6)	<0.001	3.2 (1.3)	0.9 (0.9)	<0.001	normal 14.7%	normal 44.1%%	0.0124
Vandenplas [18]	CMA-	3.0 (2.1)	0.9 (1.1)	<0.001	2.8 (1.2)	0.9 (0.9)	<0.001	normal 11.4%	normal 17.1%	0.527
Vandenplas [19]	CMA+ T-eCHF	n.r.	decrease 2.8 (2.4)	<0.001	n.r.	decrease 2.3 (1.3)	<0.001	normal 9.5%	normal 42.9%	0.020
Vandenplas [19]	CMA+ NT-eCHF	n.r.	decrease 1.9 (2.0)	<0.001	n.r.	decrease 2.2 (1.8)	<0.001	normal 12.5%	normal 37.5%	0.157
Dupont [20] °	CMA+	1.7 (1.1)	0.8 (0.6)	n.r.	1.6 (1.6)	0.9 (1.0)	n.r.	normal 53.3%	normal 66.7%	n.r.
Vandenplas [35]	FGIDs	2.24	1.23	n.r.	1.31	0.72	n.r.	hard/norm/soft/fluid/watery 19%/21%/36%/21%/4%	hard/norm/soft/fluid/watery 2%/17%/36%/37%/3%	n.r.
Vandenplas [37]	CMA+	median (IQR) 0 (0–2) range 0–6	median (IQR) 0 (0–1) range 0–5	<0.001	median (IQR) 0 (0–1) range 0–6	median (IQR) 0 (0–0) range 0–3	<0.001	median (IQR) 4 (2–4) range 0–6	median (IQR) 2 (0–4) range 0–6	<0.0001
Vandenplas [37]	CMA-	median (IQR) 0 (0–2) range 0–6	median (IQR) 0 (0–1) range 0–3	0.089	median (IQR) 0 (0–1) range 0–4	median (IQR) 0 (0–0.25) range 0–5	0.24	median (IQR) 2 (0–4) range 0–6	median (IQR) 0 (0–2) range 0–4	0.03
**Publication [Ref]**	**Group**	**Eczema** **Score**	**Eczema** **Score**	***p***	**Urticaria Score**	**Urticaria** **Score**	***p***	**Respiratory Score**	**Respiratory Score**	***p***
		**Mean (SD)**	**Mean (SD)**		**Mean (SD)**	**Mean (SD)**		**Mean (SD)**	**Mean (SD)**	
		**Baseline**	**After CM Elimination**		**Baseline**	**After CM Elimination**		**Baseline**	**After CM Elimination**	
Sirin Kose [26]	CMA+	median (Q1–Q3) 2 (0–3.5)	n.r.	n.r.	median (Q-Q3) 0 (0–6)	n.r.	n.r.	Median (Q1–Q3) 0 (0–5)	n.r.	n.r.
Salvatore [23]	CM-free diet responders	2.4 (2.2) ****	n.r.	n.r.	n.r.	n.r.	n.r.	0.6 (0.7) *****	n.r.	n.r.
Salvatore [23]	CM-free diet non-responders	0.6 (2.2) ****	n.r.	**** NS	n.r.	n.r.	n.r.	0.6 (0,8) *****	n.r.	***** NS
Vandenplas [14]	eWHF	2.1 (2.0)	0.8 (1.1)	n.r.	0.4 (1.6)	0 (0)	n.r.	0.8 (1.0)	0.4 (0.8)	n.r.
Vandenplas [14]	eCHF	1.8 (1.9)	1.0 (1.5)	n.r.	0.1 (0.5)	0 (0)	n.r.	0.8 (0.9)	0.4 (0.6)	n.r.
Vandenplas [17]	CMA+	n.r.	n.r.		present 15.8%	present 0%	<0.02	n.r.	n.r.	n.r.
Vandenplas [18]	CMA+	n.r.	n.r.	n.r.	n.r.	n.r.	n.r.	n.r.	n.r.	n.r.
Vandenplas [18]	CMA-	n.r.	n.r.	n.r.	n.r.	n.r.	n.r.	n.r.	n.r.	n.r.
Vandenplas [19]	CMA+ T-eCHF	n.r.	decrease 0.8 (1.3)	<0.01	n.r.	n.r.	n.r.	n.r.	decrease 0.6 (0.7)	0.002
Vandenplas [19]	CMA+ NT-eCHF	n.r.	decrease 1.9 (1.6)	<0.01	n.r.	n.r.	n.r.	n.r.	decrease 0.6 (0.7)	0.002
Dupont [20] °	CMA+	absent 70%	absent 80%	n.r.	absent 76.7%	absent 100%	n.r.	absent 83.3%	absent 93.3%	n.r.
Vandenplas [35]	FGIDs	n.r.	n.r.	n.r.	n.r.	n.r.	n.r.	n.r.	n.r.	n.r.
Vandenplas [37]	CMA+	median (IQR) 2 (1–4) range 0–6	median (IQR) 1 (1–2) range 0–6	<0.0001	median (IQR) 0 (0–0) range 0–6	median (IQR) 0 (0–0) range 0–6	0.06	median (IQR) 1 (0–1) range 0–3	median (IQR) 0 (0–1) range 0–2	<0.0001
Vandenplas [37]	CMA-	median (IQR) 1 (1–2) range 0–6	median (IQR) 1 (0–1) range 0–5	0.017	median (IQR) 0 (0–0) range 0–6	median (IQR) 0 (0–0) range 0–6	0.57	median (IQR) 1 (0–1) range 0–2	median (IQR) 1 (0–1) range 0–1	0.0056

Legend: CM—cow’s milk; CMA—cow’s milk allergy; eCHF—extensively casein hydrolyzed formula; eWHF—extensively whey hydrolyzed formula; IQR—interquartile range; nr—not reported; NT-eCHF—non-thickened extensively casein hydrolyzed formula; Q—quartile; SD—standard deviation; T-eCHF—thickened extensively casein hydrolyzed formula; +—positive; -—negative; ° CoMiSS as a tracking tool in infants with CMA having elimination diet; comparison of group of responders and non-responders to CM free diet (*p*): * crying, ** regurgitation, *** stool consistency, **** eczema, ***** respiratory symptoms; NS—non-significant.

**Table 9:** Diagnostic properties of CoMiSS for various cut-off values [37].

**A**. Baseline CoMiSS				
**CoMiSS Cut-Off**	**Sensitivity**	**Specificity**	**PPV**	**NPV**
≥5	88.5%	33.3%	89.7%	30.6%
≥6	78.8%	51.5%	91.4%	27.0%
≥7	68.2%	57.6%	91.4%	21.6%
≥8	56.2%	60.6%	90.4%	17.4%
≥9	43.8%	69.7%	90.5%	15.9%
≥10	35.9%	69.7%	88.6%	14.2%
≥11	25.8%	81.8%	90.3%	14.4%
≥12	20.3%	87.9%	91.7%	14.4%
**B.** Baseline CoMiSS plus ≥50% reduction from baseline to Visit 2				
**CoMiSS Cut-Off**	**Sensitivity**	**Specificity**	**PPV**	**NPV**
≥5	38.1%	62.5%	87.2%	13.1%
≥6	35.3%	68.8%	88.4%	13.7%
≥7	34.4%	71.9%	89.2%	14.0%
≥8	30.2%	71.9%	87.8%	13.3%
≥9	25.6%	78.1%	88.7%	13.5%
≥10	20.5%	78.1%	86.3%	12.8%
≥11	16.3%	84.4%	87.5%	13.0%
≥12	14.0%	87.5%	88.2%	13.1%

## Data Availability

Not applicable.
